# Acute Pancreatitis as an Unusual Culprit of Diabetic Ketoacidosis in a Nondiabetic: A Case-Based Review

**DOI:** 10.1155/2023/9122669

**Published:** 2023-08-22

**Authors:** Steven Imburgio, Apurva Vedire, Harshavardhan Sanekommu, Anmol Johal, Sobaan Taj, Christopher Lesniak, Arman Mushtaq

**Affiliations:** ^1^Jersey Shore University Medical Center, Department of Medicine, 1945 NJ-33, Neptune City, NJ 07753, USA; ^2^Jersey Shore University Medical Center, Department of Endocrinology, 1945 NJ-33, Neptune City, NJ 07753, USA

## Abstract

Acute pancreatitis has been associated with a multitude of complications including pancreatic necrosis, pseudocysts, abscesses, acute respiratory distress syndrome, disseminated intravascular coagulation, and hyperglycemia. To date, only rare case reports have demonstrated diabetic ketoacidosis (DKA) as a rare sequela of acute pancreatitis. We report a case of a 34-year-old female with no prior history of diabetes who was subsequently diagnosed with DKA after presenting with severe acute pancreatitis. This case serves as a framework to not only highlight DKA as a rare complication of acute pancreatitis but also to explore the potential pathophysiology that underlies this phenomenon including stress hyperglycemia and post-pancreatitis diabetes mellitus.

## 1. Introduction

Acute pancreatitis has emerged as the leading cause of gastrointestinal-related hospitalizations with approximately 300,000 emergency department visits in the United States each year [1]. Recent epidemiology trends over the past five decades point towards increasing frequency in reported cases [2]. Patients will classically report sharp epigastric pain that radiates to the back, along with associated symptoms of nausea, vomiting, and decreased appetite [3]. The underlying pathogenesis in acute pancreatitis is a result of autodigestion of the pancreatic parenchyma by endogenous enzymes [4]. According to the 2012 Revised Atlanta Classification, at least 2 of the following 3 criteria must be met to confirm the diagnosis: (1) abdominal pain that is characteristic of pancreatitis, (2) a lipase or amylase level that is greater than three times the upper limit of normal, and (3) abdominal imaging that shows findings of an acutely inflamed pancreas [5]. Treatment typically consists of aggressive intravenous fluid resuscitation, adequate pain management, advancing diet as tolerated, and reversing the underlying etiology if possible [6]. Together, gallstones and alcohol abuse account for an overwhelming majority of acute pancreatitis cases [7]. More rare etiologies involve hypertriglyceridemia, drug-induced, trauma, hypercalcemia, post-procedural, congenital abnormalities, toxins, and autoimmune [8]. The clinical spectrum of presentations varies from mild cases that can be treated in a conservative manner to severe disease with complications that carry high rates of morbidity and mortality [9]. Acute pancreatitis can result in a number of both localized and systemic complications including the development of pancreatic necrosis, pseudocysts, abscesses, acute respiratory distress syndrome, disseminated intravascular coagulation, and hyperglycemia [10]. We present a rare case of acute pancreatitis-induced diabetic ketoacidosis in a nondiabetic patient and review the potential etiologies of this complication.

## 2. Case Presentation

A 34-year-old female with a past medical history significant for obesity, hypertension treated with amlodipine besylate 5 mg/day, alcohol use disorder, and multiple previous hospitalizations for alcoholic pancreatitis presented with abdominal pain of 3 days duration. The pain was described as severe, epigastric in nature with radiation to the back, and similar to that of her previous pancreatitis episodes. Additionally, the patient endorsed nausea, nonbilious and nonbloody vomiting, and decreased appetite during this time. She denied any trauma, substance use, medication changes, exposure to systemic glucocorticoids, or sick contacts. Her last alcoholic beverage was several weeks prior to admission.

In the emergency department, the patient was afebrile, had heart rate of 111 beats per minute, was normotensive, and had normal oxygen saturation. In the workup of her abdominal pain, a lipase was ordered which resulted at 1,137 U/L (Ref: 12–53 U/L). Additional labs were significant for a blood glucose level of 220 (Ref: 70–99 mg/dL) and potassium of 5.2 mmol/L (Ref: 3.5–5.1 mmol/L). The patient's initial lab values are listed in Table 1. Anion gap at this time was 13 (Ref: 5–15 mmol/L). Urine drug screen was negative for any illicit substance, and her blood alcohol percentage on admission was normal at 0.003% (Ref: < 0.005%). A computed tomography scan of the abdomen/pelvis demonstrated pancreatic and peripancreatic edema consistent with acute pancreatitis. Based on the patient's characteristic abdominal pain, elevated lipase level, and imaging findings, she was subsequently admitted for acute pancreatitis. Despite multiple episodes of acute pancreatitis, the patient did not show signs of chronic pancreatitis given the absence of pancreatic calcifications on imaging and lack of exocrine dysfunction such as diarrhea or nutritional deficiencies. She received moderate fluid resuscitation with a 1-liter bolus of lactated Ringer's solution followed by a maintenance rate of 150 mL/hour. Additionally, she was initially kept nothing by mouth (NPO) due to vomiting, while her pain was managed with intravenous medications.

Further workup of the etiology of the patient's acute pancreatitis was performed. The patient's calcium and triglyceride levels were 10.0 mg/dL (Ref: 8.7–10.4 mg/dL) and 183 mg/dL (Ref: 0–150 mg/dL), respectively. A right upper quadrant ultrasound revealed no evidence of cholelithiasis or bile duct dilation. Home medications were also reviewed with no clear pharmacological source of the patient's pancreatitis detected as she was only taking amlodipine besylate 5 mg/day for hypertension.

Approximately 8 hours after arriving at the hospital, the patient's blood sugars rapidly rose to a peak of 550 mg/dL. At this time, she was found to have a high anion gap metabolic acidosis with an anion gap of 17.0 mmol/L and a venous blood gas showing a pH of 7.132 (Ref: 7.320–7.420) and bicarbonate of 6.4 (Ref: 24.0–26.0 mmol/L). Due to concern of diabetic ketoacidosis, a stat beta-hydroxybutyrate level was ordered which resulted as >4.50 (Ref: 0.02–0.27 mmol/L). The patient was immediately transferred to the medical intensive care unit (ICU) for higher level of care with an insulin drip started. The endocrinology team was consulted for management of her new onset diabetic ketoacidosis, and the following day she was successfully converted to a basal/bolus insulin regimen.

After being downgraded from the ICU to the medical floors, the patient's HbA1C came back at 4.5% which did not correlate to such abnormally high blood sugar levels and suggested an acute rapid elevation. An extensive chart review revealed no previous listed diagnosis of diabetes and a previous HbA1C measurement of 5.5% three years prior. The initial C-peptide levels were decreased at 0.11 ng/mL (Ref: 0.80–3.85 ng/mL) and were then repeated two days later and resulted as 0.30 ng/mL suggesting decreased levels of endogenous insulin production. Glutamic acid decarboxylase-65 antibody levels were <5 IU/mL (Ref: <5 IU/mL). Other antibody levels were not checked. Despite resolution of the acute pancreatitis episode, the patient had persistent hyperglycemia requiring insulin glargine 20 units nightly, lispro insulin 6 units before meals, and moderate lispro insulin sliding scale to maintain blood sugar levels below 200 mg/dL. After this comprehensive workup, she was ultimately diagnosed with diabetic ketoacidosis from a multifactorial etiology including an exaggerated initial stress hyperglycemic response in the setting of her critical illness state and prolonged elevated blood sugar levels concerning for possible post-pancreatitis diabetes mellitus given her prior history of recurrent pancreatitis. The patient had an uncomplicated remaining hospital course and was safely discharged with insulin. She was scheduled for close outpatient follow-up with her primary care provider and endocrinology.

## 3. Discussion

DKA is a consequence of inadequate insulin activity and as a result primarily occurs in type 1 diabetes mellitus patients [11]. DKA is potentially life-threatening and often necessitates ICU management for volume resuscitation, insulin drip, and frequent monitoring of electrolyte and glucose levels [12]. A possible complication of DKA is the development of moderate to severe hypertriglyceridemia due to increased levels of lipolysis in adipose tissue in response to insulin deficiency [13]. Hyperglyceridemia is responsible for approximately 10% of all cases of acute pancreatitis with a review of 31,740 patients demonstrating that the risk of development increases in a direct relationship to triglyceride concentration [14]. Most cases of acute pancreatitis occur when serum triglycerides levels surpass 1,000 mg/dL [15]. Infrequently, the acute elevation of triglycerides that sometimes occurs in DKA can trigger the development of acute pancreatitis. The proposed mechanism involves increased plasma viscosity due to the elevated triglyceride levels, predisposing the pancreatic parenchyma to organ inflammation and ischemic damage [16]. This triad of DKA and hypertriglyceridemia along with the subsequent development of acute pancreatitis has been described by multiple case reports [17–20].

While acute pancreatitis occurring from the profound hypertriglyceridemia of DKA has been well documented in the literature, the reverse phenomenon of acute pancreatitis presenting first followed by the development of DKA with no dramatic rise in triglyceride levels is scarcely described and even more poorly understood. The primary proposed mechanism of post-pancreatitis DKA likely involves multiple stress-mediated physiologic pathways contributing to a state of acute hyperglycemia [21]. One proposed hypothesis of stress hyperglycemia involves a neuroendocrine response to a critical illness like acute pancreatitis that results in subsequent activation of the hypothalamic-pituitary-adrenal axis [22]. Acute changes in adrenocorticotropic hormone levels can increase serum cortisol levels, stimulating hepatic gluconeogenesis and inhibiting the uptake of glucose in peripheral tissues [23]. This effect may be compounded by activation of the sympathetic nervous system and presence of pro-inflammatory cytokines which have both been reported in acute pancreatitis [24, 25]. The resulting elevation in epinephrine and norepinephrine increases circulating glucose levels through hepatic gluconeogenesis and glycogenolysis [22]. Pro-inflammatory cytokines, which have a synergistic effect in addition to adrenergic agonists, can produce profound elevation in serum glucose levels through an increase in peripheral insulin resistance [26]. Stress hyperglycemia involves a complex multifactorial etiology that causes a rapid elevation in serum glucose levels, in a manner similar to that of exogenous corticosteroids, which may provoke DKA in patients undergoing acute pancreatitis who are inherently predisposed to insulin intolerance.

Additionally, a concept known as post-pancreatitis diabetes mellitus has emerged in the literature which can serve as another etiology for DKA after an episode of acute pancreatitis. The pathophysiology is fairly straightforward with decreased insulin production due to inflammation that results in progressive fibrosis of the exocrine tissue [27]. This phenomenon can be differentiated by the presence of prolonged hyperglycemia which is not seen in stress hyperglycemia [28]. Unlike our case, the transient rise of blood sugar levels from isolated stress hyperglycemia is expected to resolve spontaneously after addressing the underlying acute illness. While there are no established guidelines for screening of post-pancreatitis diabetes mellitus, this entity should be suspected in any adult who meets the diagnostic criteria for diabetes by the American Diabetes Association following an episode of pancreatitis [29]. Current data point to a 2-fold increase in the risk of developing diabetes mellitus following a single episode of acute pancreatitis [30]. Previous studies have also demonstrated that recurrent pancreatitis is associated with a higher risk of developing diabetes mellitus [31]. These findings suggest that individuals with repetitive episode of pancreatitis, such as our patient, may be predisposed to hyperglycemia through decompensated function of the pancreatic *β*-cells.

Understanding the pathogenesis of DKA-induced acute pancreatitis is important as this disease state carries significant differences in treatment and prognosis as compared to acute pancreatitis alone. Appropriate management includes fluid resuscitation, along with specific management of the DKA with intravenous insulin and electrolyte repletion, in conjunction with the traditional therapeutic approach for acute pancreatitis [32]. Having a high clinical suspicion for DKA as a possible complication is necessary as abdominal pain is a classic overlapping symptom, and false anchoring bias on the initial acute pancreatitis diagnosis may result in delayed treatment of the acidosis and dehydration, thus leading to possible severe adverse outcomes [33]. Apart from the obvious differences in treatment of these distinct conditions, the clinical significance of recognizing acute pancreatitis as a complication of DKA stems from the fact that this patient demographic has worse clinical outcomes as compared to patients with acute pancreatitis without concurrent DKA. To highlight this point, Simons-Linares et al., after analyzing 33,356 patients with coexisting acute pancreatitis and DKA, found longer hospital length of stays, increased inpatient mortality, and higher complication rates of acute kidney injury, ileus, acute respiratory distress syndrome, and sepsis as compared to patients with acute pancreatitis in the absence of DKA [34]. An important consideration is that this study did not assess the incidence of pre-existing diabetes among the two groups. Based on these findings, acute pancreatitis patients may benefit from more frequent glucose monitoring and strict adherence to inpatient blood sugar goals to prevent DKA from developing as a dangerous sequela. However, there are currently no enough data due to the rarity of this condition to confidently recommend a benefit in routine testing of ketones or bicarbonate in hospitalized patients with acute pancreatitis.

## 4. Conclusion

DKA and acute pancreatitis are two well-known disease processes that are often seen in the hospital setting. While acute pancreatitis has been reported as a complication of DKA when profound hypertriglyceridemia is present, we describe a rare case of acute pancreatitis preceding the development of DKA in a patient without significantly elevated serum triglycerides and no prior history of diabetes mellitus. We aim to delineate a rare etiology of DKA as a complication of acute pancreatitis. Through this case-based review, we explore the possible pathophysiology of this phenomenon through mechanisms that include stress hyperglycemia and post-pancreatitis diabetes mellitus. A high degree of clinical suspicion is warranted in cases of acute pancreatitis for the emergence of DKA as this condition may be difficult to detect due to overlap in symptoms. Additionally, close monitoring of labs for DKA development is important since missing this diagnosis in patients with acute pancreatitis may lead to significantly worse clinical outcomes.

## Figures and Tables

**Table 1:** Initial laboratory values.

Labs	Value (unit)	Reference range
White blood cells	8.2 10^3^/uL	4.5–11.0 10^3^/uL
Red blood cells	4.45 10^6^/uL	4.10–5.10 10^6^/uL
Hemoglobin	15.5 g/dL	12–16 g/dL
Hematocrit	48.4%	35.0–48.0%
Platelet count	193 10^3^/uL	140–450 10^3^/uL
Glucose	220 mg/dL	70–99 mg/dL
Blood urea nitrogen	27 mg/dL	9–23 mg/dL
Creatinine	0.94 mg/dL	0.55–1.02 mg/dL
Sodium	137 mmol/L	136–145 mmol/L
Potassium	5.2 mmol/L	3.5–5.1 mmol/L
Chloride	95 mmol/L	98–107 mmol/L
Anion gap	13 mmol/L	5–15 mmol/L
Calcium	10.0 mg/dL	8.7–10.4 mg/dL
Carbon dioxide	21 mmol/L	20–31 mmol/L
Alkaline phosphatase	96 U/L	46–116 U/L
Albumin	4.7 g/dL	3.4–5.0 g/dL
Total bilirubin	0.9 mg/dL	0.2–1.3 mg/dL
AST	50 U/L	0–34 U/L
ALT	22 U/L	10–49 U/L
Lipase	1,137 U/L	12–53 U/L
Triglycerides	183 mg/dL	0–150 mg/dL

## Data Availability

All data generated or analyzed during this study are included in this article. Further inquiries can be directed to the corresponding author.
